# Impact of the Seattle Sweetened Beverage Tax on substitution to alcoholic beverages

**DOI:** 10.1371/journal.pone.0262578

**Published:** 2022-01-18

**Authors:** Lisa M. Powell, Julien Leider

**Affiliations:** 1 Division of Health Policy and Administration, School of Public Health, University of Illinois Chicago, Chicago, IL, United States of America; 2 Institute for Health Research and Policy, University of Illinois Chicago, Chicago, IL, United States of America; Universita degli Studi di Brescia, ITALY

## Abstract

**Introduction:**

Taxes are increasingly used as a policy tool aimed at reducing consumption of sugar-sweetened beverages (SSBs), given their association with adverse health outcomes including type 2 diabetes, obesity and cardiovascular disease. However, a potential unintended consequence of such a policy could be that the tax induces substitution to alcoholic beverages. The purpose of this study is to examine the impact of the $0.0175 per ounce Seattle, Washington, Sweetened Beverage Tax (SBT) on volume sold of alcoholic beverages.

**Methods:**

A difference-in-differences estimation approach was used drawing on universal product code-level food store scanner data on beer (N = 1059) and wine (N = 2655) products one-year pre-tax (February-November, 2017) and one and two-years post-tax (February-November, 2018 and 2019) with Portland, Oregon, as the comparison site.

**Results:**

At two-years post-tax implementation, volume sold of beer in Seattle relative to Portland increased by 7% (ratio of incidence rate ratios [RIRR] = 1.07, 95% CI:1.00,1.15), whereas volume sold of wine decreased by 3% (RIRR = 0.97, 95% CI:0.95,1.00). Overall alcohol (both beer and wine) volume sold increased in Seattle compared to Portland by 4% (RIRR = 1.04, 95% CI:1.01,1.07) at one-year post-tax and by 5% (RIRR = 1.05, 95% CI:1.00,1.10) at two-years post-tax. The implied SSB cross-price elasticities of demand for beer and wine, respectively, were calculated to be 0.35 and -0.15.

**Conclusions:**

There was evidence of substitution to beer following the implementation of the Seattle SSB tax. Continued monitoring of potential unintended outcomes related to the implementation of SSB taxes is needed in future tax evaluations.

## Introduction

Sugar-sweetened beverage (SSB) taxes are fiscal policy tools, similar to taxes on alcohol and tobacco products, that can raise revenue and promote health [1]. The public health case for SSB taxes is to reduce demand, given that SSB consumption is linked to adverse health outcomes such as type 2 diabetes, obesity and cardiovascular disease [2–5]. Taxes change relative prices of taxed versus untaxed products which, in turn, impacts choices regarding consumption. The key mechanism through which this occurs is via an increase in the price of the taxed products, known as tax pass-though, which reduces the quantity demanded of those products, all else constant. Indeed, a number of evaluations of sweetened beverage taxes in the U.S. have found associations with reduced demand [6–13]. These are effectively own-price effects and are typically quantified in terms of the own-price price elasticity of demand, the percentage change in quantity demanded as a result of a one percent change in price. Both demand models and tax evaluations have estimated the demand for SSBs to be price sensitive (with price elasticities in absolute value generally ≥ 1) [12, 14–16].

Given that taxes change relative prices of taxed and untaxed products, we also expect there to be cross-price effects. In particular, if some beverage types (e.g., those with added sugars) are taxed but others (e.g., diet drinks, water, milk, 100% juice) are not, then we would expect potential increases in demand for untaxed beverages if consumers view them as substitutes. Tax evaluations to date of both U.S. local taxes and national taxes worldwide have mostly focused on assessing substitution to non-alcoholic beverages and have generally found consistent evidence of some substitution, particularly to bottled water [6–9, 12, 17, 18]. Notably, however, some evaluations of the Philadelphia, PA, and Cook County, IL, taxes, which applied to both SSBs and artificially sweetened beverages, found no evidence of substitution to untaxed non-alcoholic beverages [11, 13].

Beyond substitution to untaxed non-alcoholic beverages, a potential unintended consequence of a SSB tax could be substitution to alcoholic beverages. This would be problematic if it contributed to excess alcohol consumption, which has been shown to be associated with higher risk of motor accidents/deaths, liver cirrhosis, sexually transmitted diseases, crime and violence, and workplace accidents [19]. With respect to changes in own-prices, numerous studies have examined the demand for alcohol and found that demand is responsive to changes in own-prices albeit being less price sensitive than SSBs (with own-price elasticities of demand for beer, wine and spirits generally < 1) [20–22]. However, limited and mixed evidence exists on the extent of cross-price effects of changes in relative prices between SSBs and alcohol. Evidence from a demand system model based on UK data suggests that high-sugar SSBs and beer are substitutes while high-sugar SSBs and spirits are complements, with no significant cross-price effects between high-sugar SSBs and wine; while medium-sugar SSBs are found to be complements with beer and wine, but substitutes for spirits [23]. Evidence from another demand system model drawing on data from Vietnam finds significant cross-price effects suggesting that both beer and wine are substitutes for SSBs (the study did not assess spirits) [24]. A randomized controlled trial that increased the prices of SSBs in a virtual supermarket setting (to simulate a Dutch value-added tax increase from 6% to 19% on SSBs), found that while participants reduced purchases of the taxed SSBs, there were no changes in overall purchases of alcoholic drinks [25]. A recent evaluation of the Philadelphia, PA, sweetened beverage tax found that the tax was not associated with changes in volume sold of wine or spirits; however, the tax evaluation did not assess potential substitution to beer [26].

Previous analyses of the $0.0175 per ounce Seattle, Washington, Sweetened Beverage Tax (SBT), which was implemented on January 1, 2018, found that at both one-year and two-years post-implementation the volume sold of taxed beverages fell by 22% with no offset from cross-border shopping, and that there was some moderate substitution to untaxed non-alcoholic beverages, of which volume sold increased by 4% at one-year post-tax and by 5% at two-years post-tax [12, 27]. This study extends that work to examine one- and two-year post-tax implementation changes in volume sold of alcoholic beverages (beer and wine) in the same set of stores (supermarkets and grocery, drug, convenience, dollar, and mass merchandise stores). That is, we aim to assess the extent to which higher prices for SSBs induced shoppers to switch to purchases of alcoholic beverages. We are particularly interested in substitution to beer as it has lower alcohol content compared to wine and spirits and, therefore, may be more likely to serve as a substitute for SSBs. This is the first study to our knowledge to empirically assess the extent to which the implementation of a SSB tax may lead to substitution to beer. We use a difference-in-differences (DID) study design drawing on weekly store scanner data to assess the impact of the Seattle SBT on changes in volume sold of beer and wine up to two-years post-tax implementation relative to changes in volume sold in Portland, Oregon.

## Methods

### Data and measures

The data used in this study are universal product code (UPC)-level retail scanner data obtained from Nielsen on weekly units and dollar amounts sold of alcoholic beer (defined to include flavored malt beverages and ciders) and wine beverages in grocery, drug, convenience, dollar, and mass merchandise stores, and supermarkets in the intervention site of Seattle, Washington, and the comparison site of Portland, Oregon. These data are from the same source as those previously used to examine the impact of the Seattle SBT on non-alcoholic beverages and are described in more detail elsewhere [12, 27]. As described previously, Portland was chosen as the comparison site out of eight municipalities, including the four largest municipalities in both Washington and Oregon, based on Mahalanobis distance matching on key sociodemographic and socioeconomic characteristics, including population size, median household income, the percentage of the population below 125% of the poverty line, the percentage that was non-Hispanic black or Hispanic, and the percentage that was non-Hispanic Asian [12].

**Fig 1:**
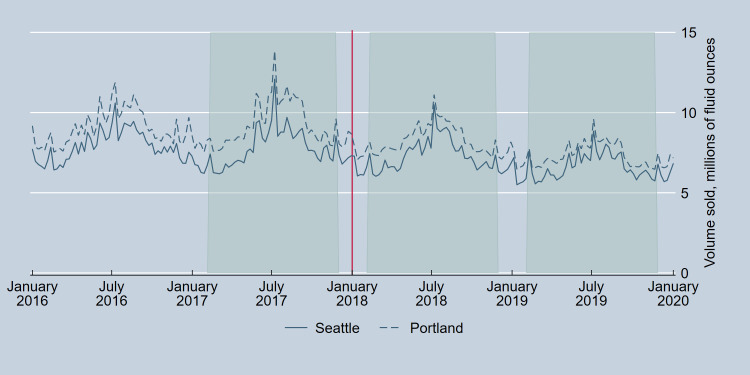
Volume sold of beer in Seattle, Washington, and Portland, Oregon, pre- and post-implementation of the Seattle Sweetened Beverage Tax. The vertical line indicates the date of tax implementation. Shaded regions correspond to the analytical time periods.

**Fig 2:**
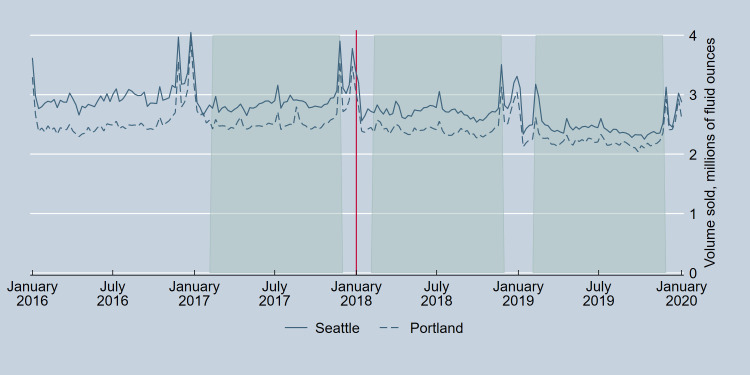
Volume sold of wine in Seattle, Washington, and Portland, Oregon, pre- and post-implementation of the Seattle Sweetened Beverage Tax. The vertical line indicates the date of tax implementation. Shaded regions correspond to the analytical time periods.

Parallel trends between Seattle and Portland in weekly total volume sold of beer and wine per capita [28] in the pre-tax period were tested with Wald tests for the joint significance of site by month interactions in linear regression models with robust standard errors. This test failed for wine (p = 0.009) although not beer (p = 0.44) when considering the entire one-year pre-tax period from January 8 to December 30, 2017, but passed for both wine (p = .27) and beer (p = .27) when restricting the period to February 5 to November 25, 2017. As a result, analyses compared this pre-tax period to the same 42-week period in 2018 and 2019, one- and two-years post-tax. Figs 1 and 2 provide further visual evidence of parallel trends for the analytical sample.

The analytical samples excluded a small number of UPCs (representing less than 0.1% of total units sold for both beer and wine) for which the per-unit volume necessary for computing total volume sold was not available, and were balanced to only include beer and wine UPCs sold in both Seattle and Portland in all three years, leaving 1059 beer and 2655 wine UPCs. These UPCs represented 83.7% and 91.5% of the original volume sold of beer and wine, respectively, including similar percentages over time: 87.8%, 85.0%, and 78.0% of the original volume sold of beer and 93.2%, 92.5%, and 88.6% of the original volume sold of wine in the pre-tax, one-year post-tax, and two-year post-tax periods, respectively. This held similarly by site and time, as the balanced sample of UPCs included 86.8%, 85.0%, and 77.6% in Seattle and 88.6%, 85.1%, and 78.4% in Portland of the original volume sold of beer in the pre-tax, one-year post-tax, and two-year post-tax periods, respectively; and, 93.3%, 92.7%, and 87.8% in Seattle and 93.1%, 92.4%, and 89.4% in Portland of the original volume sold of wine in those periods. The total volume sold in fluid ounces of each UPC was computed in each combination of site and year.

### Statistical analyses

DID analyses were conducted to assess changes in volume sold of beer and wine from one-year pre-tax to one-year and two-years post-tax in Seattle relative to changes in the comparison site of Portland. Specifically, separate models for beer and wine were estimated of the following form:

$$E(Y_{ist}|{Seattle}_{s},\;{1YRPost}_{t},\;{2YRPost}_{t})=exp\;(\beta_{0}+\beta_{1}{Seattle}_{s}+\beta_{2}{1YRPost}_{t}+\beta_{3}{2YRPost}_{t}+\beta_{4}{Seattle}_{s}∙{1YRPost}_{t}+\beta_{5}{Seattle}_{s}∙{2YRPost}_{t})$$

where *Y*_*ist*_ is the outcome of either beer or wine total volume sold for UPC *i* in site *s* and time period *t*; *Seattle*_*s*_ is a binary indicator for observations in the intervention site of Seattle; and *1YRPost*_*t*_ and *2YRPost*_*t*_ are binary indicators for observations one-year and two-years post-tax implementation, respectively. A model was also run including both beer and wine; this model included an additional indicator *Beer*_*i*_ for observations of beer UPCs. Poisson DID regressions were used to model changes in volume sold because the effect of the tax on volume sold was expected to be multiplicative rather than additive (i.e., a percentage change rather than a fixed number of ounces) [29]. The exponentiated coefficients from these regressions are incidence rate ratios, and the exponentiated DID coefficient is a ratio of incidence rate ratios (RIRR) showing the percentage change in volume sold in Seattle relative to Portland. Models were computed for aggregated yearly total volume sold at the UPC level in order to allow the estimation of average year-over-year changes in volume sold across UPCs between the pre-tax and one-year and two-year post-tax periods, respectively. The models were estimated with robust standard errors clustered on UPC. Analyses were conducted in Stata/SE 15.1 (StataCorp LP, College Station, TX). This study was approved by the University of Illinois Chicago Institutional Review Board.

## Results

Table 1 presents summary statistics of the volume sold of beer, wine, and total alcohol (beer and wine) in Seattle and Portland in the pre-tax, one-year post-tax, and two-year post-tax periods, on average across UPCs. Pre-tax in Seattle, the total volume sold of beer (based on the mean times the number of UPCs) was almost three times that of wine (332 million versus 121 million ounces). In Seattle, mean volume sold of beer, wine, and total alcohol, respectively, was 313,529, 45,390 and 121,847 ounces pre-tax implementation, and at two-years post-tax mean volume sold had declined to 265,061, 38,623, and 103,189 ounces. In Portland, the mean volume sold of beer, wine, and total alcohol, respectively, was 373,053, 39,988 and 134,957 ounces pre-tax implementation and had declined to 293,994, 34,988, and 108,840 ounces at two-years post-tax.

**Table 1:** Mean alcoholic beverage volume sold in Seattle, Washington, and Portland, Oregon, before and after implementation of the Seattle Sweetened Beverage Tax, 2017–2019^a^.

Site/Time Period	Beer (N = 1,059)	Wine (N = 2,655)	Total, Beer and Wine (N = 3,714)
	Volume sold, fluid ounces, mean (SD)		
**Seattle**			
Pre-Tax	313,529 (824,506)	45,390 (108,750)	121,847 (465,640)
Year 1 Post-Tax	294,363 (779,665)	43,236 (103,581)	114,842 (440,158)
Year 2 Post-Tax	265,061 (796,556)	38,623 (94,926)	103,189 (444,627)
**Portland**			
Pre-Tax	373,053 (876,164)	39,988 (106,735)	134,957 (499,502)
Year 1 Post-Tax	333,861 (811,407)	38,171 (103,029)	122,483 (461,537)
Year 2 Post-Tax	293,994 (739,535)	34,988 (97,464)	108,840 (419,890)

^a^ Total volume sold was computed for each universal product code (UPC) in each site and time period. Means and SDs were computed across UPCs. The pre-tax period included February 5, 2017 through November 25, 2017, and the year 1 and year 2 post-tax periods included the same 42 weeks in 2018 and 2019, respectively. Sample sizes shown are in terms of UPCs.

The DID results for changes in volume sold of beer, wine, and total alcohol (both beer and wine) in Seattle relative to Portland from the pre-tax period to one-year and two-years post-tax implementation are presented in Table 2. For beer, the results show that at one-year post-tax implementation volume sold increased by 5% (RIRR = 1.05, 95% CI: 1.01, 1.09) and that this impact was sustained and was slightly higher at two-years post-tax implementation with an increase of 7% (RIRR = 1.07, 95% CI: 1.00, 1.15). Based on a Wald test, there was no statistically significant difference between the impacts for beer at one-year and two-years post-tax implementation (p = 0.29). There were no statistically significant changes in volume sold of wine one-year post-tax implementation. However, two-years post-tax volume sold of wine decreased by 3% (RIRR = 0.97, 95% CI: 0.95, 1.00) in Seattle relative to Portland, and this differed significantly from the impact at one-year post-tax (p = 0.003). Based on a DID model including additional interaction terms with an indicator for beer, volume sold of beer increased relative to volume sold of wine in Seattle relative to Portland at both one-year post-tax (RIRR = 1.05, 95% CI: 1.01, 1.10) and two-years post-tax (RIRR = 1.10, 95% CI: 1.03, 1.19). Overall alcohol (both beer and wine) volume sold increased in Seattle compared to Portland by 4% (RIRR = 1.04, 95% CI: 1.01, 1.07) at one-year post-tax and by 5% (RIRR = 1.05, 95% CI: 1.00, 1.10) at two-years post-tax, driven by the increases in volume sold of beer.

**Table 2:** Difference-in-differences estimates of the impact of the Seattle, Washington, sweetened beverage tax on volume sold of alcoholic beverages in Seattle relative to Portland, Oregon, 2017–2019^a^.

Alcoholic Beverage Type	Year 1 Post-Tax	Year 2 Post-Tax
	RIRR (95% CI)	RIRR (95% CI)
Beer (N = 1,059)	1.05 (1.01–1.09)	1.07 (1.00–1.15)
Wine (N = 2,655)	1.00 (0.98–1.02)	0.97 (0.95–1.00)
Total, Beer and Wine (N = 3,714)	1.04 (1.01–1.07)	1.05 (1.00–1.10)

Abbreviations: RIRR, ratio of incidence rate ratios.

^a^ RIRRs were computed from Poisson regression models with robust standard errors clustered on universal product code (UPC). The model for total volume sold including both beer and wine controlled for whether the UPC was beer or wine. The pre-tax period included February 5, 2017 through November 25, 2017, and the year 1 and year 2 post-tax periods included the same 42 weeks in 2018 and 2019, respectively; the estimates shown are for the tax impact in years 1 and 2 post-tax relative to the pre-tax period. Sample sizes shown are in terms of UPCs; the number of observations for estimation across the two sites and three time periods was equal to six times the number of UPCs.

## Discussion

This study found that following the implementation of the Seattle SBT, there was a sustained increase in the volume sold of beer in Seattle relative to the comparison site of Portland, reflected in a 5% and 7% increase in the respective one- and two-year post-tax periods. At one-year post-tax there was no significant change in volume sold of wine, but in the two-year post-tax period volume sold of wine in Seattle relative to Portland fell slightly by 3%. In terms of overall changes in total alcohol sold, measured in this study by beer and wine, the decrease in volume sold of wine partly offset the larger increase in volume sold of beer in year two with a net increase of 5% in total alcohol volume sold in the second year following the implementation of the SBT.

The result in this study of a significant sustained increase in volume sold of beer following the implementation of the Seattle SBT suggests that SSBs and beer are substitutes. Drawing on previous estimates that the Seattle SBT led to an estimated 20% increase in taxed SSB prices [27], the back-of-the envelope implied cross-price elasticity of demand between SSBs and beer is 0.35 at two-years post-tax. The result from this study that SSBs and beer are found to be substitutes is consistent with previous results based on a demand system which estimated a cross-price elasticity between SSBs and beer of 0.25 [24]. The results are also consistent with another demand system study that found that high-sugar SSBs and lager are substitutes with an estimated cross-price elasticity of demand of 0.23; although the same study found that medium-sugar SSBs and beer and lager are complements with respective cross-price elasticities of -0.12 and -0.13 [23].

With respect to wine, drawing on previously estimated changes in SSB prices and the longer-run two-year post-tax results from our study, the implied cross-price elasticity of demand between SSBs and wine is -0.15, suggesting that SSBs and wine are complements. The existing literature on the extent of complementarity versus substitutability of SSBs and wine is mixed. Our results are consistent with the demand system study finding that medium-sugar SSBs and wine are complements (with an estimated cross-price elasticity of -0.03), but differ to that same study’s finding of no cross-price association between high-sugar SSBs and wine [23]. Additionally, our results differ from those of another demand system study finding that SSBs and wine are substitutes with an estimated cross-price elasticity of 0.17 [24]. Of note, our one-year post-tax finding of no change in volume sold of wine following the implementation of the Seattle SBT is consistent with the null effect found for one-year post-tax changes in volume sold of wine in a recent evaluation of the Philadelphia sweetened beverage tax [26]. The Philadelphia tax evaluation did not assess impacts beyond one-year post-tax implementation and, thus, we are unable to compare our two-year study finding of lower volume sold of wine.

This study is subject to several limitations. First, our examination of substitution to alcohol is limited to beer and wine and does not include spirits. Second, our store scanner data are limited to the same set of stores assessed for non-alcoholic beverages. However, it must be acknowledged that both Seattle and Portland have “liquor” stores that sell beer, wine and liquor which we do not capture, and we do not have information on the proportion of beer and wine sold in food stores out of total stores. Thus, it is important to keep in mind that this study only assesses the extent of changes in beer and wine sold in food stores following the implementation of the SBT. Third, we do not capture alcohol sales from bars and restaurants, which could be important. Fourth, changes were assessed based on data from store sales and, thus, we could not examine changes in individual-level consumption patterns, particularly with respect to potential changes in excessive alcohol consumption. Fifth, due to confidentiality restrictions, our store scanner data are aggregated at the site level, and we do not have access to store-level data to be able to account for store type or other store-level characteristics.

Despite these limitations, this study contributes to the understanding of potential unintended impacts from SSB tax policy in terms of substitution to alcoholic beverages and, to our knowledge, it is the first study to examine substitution to beer following the implementation of a SSB tax. The study results show that there was a sustained 5–7% increase in volume sold of beer in food stores in Seattle relative to Portland following the implementation of the Seattle SBT. However, while this study found a moderate increase in the volume sold of beer, as noted above, we were unable to assess the extent to which on the margin this may have contributed to increases in excessive alcohol consumption and consumption-related harms. A substantial body of literature has shown that higher taxes and prices for alcohol are associated with reductions in the harmful consequences of excessive drinking [20, 21]. To better understand the potential impact of the unintended consequence of substitution to beer found in this study, future studies should examine the extent of changes in adverse health outcomes and other harms (e.g., accidents, violence) linked to excessive alcohol consumption.

## Conclusions

Given the evidence found in this study on substitution to beer in food stores following the implementation of the Seattle SBT, policymakers should take into consideration these potential unintended consequences in implementation plans for future SSB taxes. As part of this process, it is important to generate further evidence on the extent of potential increases in excessive drinking and any associated harms. To address such potential unintended consequences, policymakers may consider implementing not only public health campaigns that highlight harms of added sugars in SSBs but also parallel campaigns that address harms associated with excessive drinking. The results from this study highlight the importance of continued monitoring of potential unintended outcomes related to the implementation of SSB taxes in future tax evaluations.
